# Preparation and Magnetic Properties of Low-Loss Soft Magnetic Composites Using MgO-Phenolic Resin Coating

**DOI:** 10.3390/ma17164039

**Published:** 2024-08-14

**Authors:** Lirui Wan, Xiaoran Sun, Jiechao Li, Shen Wu

**Affiliations:** 1 Institute of Electromechanical Engineering and Intelligent Manufacturing, Zhengzhou Vocational College of Information Technology, Zhengzhou 450008, China; xingchen778899@126.com; 2 Institute of Mechanical and Electrical Engineering, Zhengzhou University of Light Industry, Zhengzhou 450002, China; 18240529554@126.com (X.S.); ljczzuli@126.com (J.L.)

**Keywords:** soft magnetic composites, insulation coating, core loss

## Abstract

Optimizing the interface between a magnetic powder matrix and an oxide-insulating layer is an effective method to improve the permeability and lower eddy current loss of iron-based soft magnetic composites. In this study, in order to improve the bonding strength of the substrate and insulation layer, soft magnetic composites were prepared by pressing and heat treating with reduced iron powder as a magnetic matrix, high-temperature MgO nanoparticles as insulating coating, and phenolic resin as an adhesive. The effects of MgO content on the microstructure and magnetic properties of the composites were investigated. The results of a scanning electron microscopy and an energy-dispersive spectrometer analysis corroborate that the results obtained regarding the frequency characteristics and the resistivity of the iron powder agree with the scanning electron microscope (SEM) and energy dispersive spectrometer (EDS) analysis and confirm their improvement by the presence of an insulating layer of MgO. The resistivity of the sample coated with 4 wt.% MgO is nearly 45 times higher than that of the uncoated sample under the same conditions. The MgO-insulating film formed on the surface of iron powder makes the coated sample have low effective grain size, high resistivity, and low magnetic loss at a high frequency. At 1 kHz, the magnetic loss of the 4 wt.% MgO-coated sample is reduced by 77.3%, and the magnetic loss is only 5.8% compared with the uncoated sample at 50 kHz. This magnetic loss separation study shows that the addition of MgO insulation material can effectively reduce the eddy current loss of the magnetic powder core. The 4 wt.% MgO-coated sample has the lowest hysteresis loss factor and relatively low eddy current loss factor, so it can be determined that the addition of 4 wt.% MgO is the optimum content to attain a low magnetic loss.

## 1. Introduction

Soft magnetic composites (SMCs, also known as magnetic powder core) are prepared by powder metallurgy after surface coating with magnetic metal particles, which have the advantages of three-dimensional isotropy, low eddy current loss, good frequency characteristics, and easy machining [1,2]. As a crucial material in power electronic power conversion equipment, they find widespread applications in energy, information, and national defense sectors. However, with the increasing demand for high-density, high-integration, high-frequency, and miniaturized electronic devices, soft magnetic composites are subjected to more rigorous performance requirements, such as high permeability, saturated magnetic induction, and low power loss [3].

The matrix of soft magnetic composite materials consists of metal magnetic particles, and, due to their low electrical resistivity, result in significant eddy current loss and substantial heat loss at high frequencies. To address this issue, researchers have proposed covering the surface of the metal magnetic particles with a high-resistivity insulation layer. This approach aims to prevent direct contact between particles, thereby reducing eddy current loss and enabling efficient application at high frequencies [4].

Insulation coating is the key factor to the preparation process of soft magnetic composite materials, which largely determines the compression density, permeability, resistivity, magnetic loss, and mechanical strength of the composite materials. Therefore, the selection of insulation coating materials, coating content and the binding effect and uniformity of insulation layer and matrix have become the focus of current research in this field. Insulation coating materials are mainly divided into organic materials and inorganic materials [5]. Compared to an organic insulating layer (epoxy resin, phenolic resin, silicone resin, etc.), the inorganic insulating layer (phosphate, oxide, nitride, etc.) has better thermal stability, which can effectively solve the problem that organic coating is difficult to carry out stress annealing under high temperature and fully release the internal stress generated during pressing and maintain the integrity of the insulation coating [6]. H. Lan et al. [7] used a chemical co-precipitation method to coat the surface of Fe powder with a SiO_2_ coating layer, forming stable Fe-O and Fe-Si covalent bonds at the Fe/SiO_2_ interface, effectively binding conduction electrons to improve surface resistivity and reduce the magnetic loss of the material. Park J-H et al. [8] prepared a uniform MgO insulation layer with a thickness of 600 nm on the surface of Fe-Ni magnetic powder using a sol–gel method. The stress removal temperature of iron-based materials is between 570 °C and 775 °C [9], and MgO has high thermal stability [10], so the MgO layer can remain intact at 800 °C, improving the thermal stability and soft magnetic properties of Fe-Ni soft magnetic composites. M. Jakubčin et al. [11] prepared ferro-based soft magnetic composites with MgO insulation contents of 2 wt.%, 3 wt.%, and 5 wt.%, respectively, and studied the relationship between the permeability of the sample and the magnetic field. The results showed that the decrease in MgO content led to the increase in the proportion of irreversible magnetization process, and the lower internal demagnetization field improved the magnetic interaction between ferromagnetic particles. The movable magnetic domain walls increased, thus facilitating magnetization reversal. These studies show that the soft magnetic composite prepared with a high-temperature-resistant oxide as the insulating layer has excellent thermal stability and magnetic properties. However, the oxide is brittle, and it is easy to break during the pressing process, resulting in direct contact with the metal matrix particles, which reduces its resistivity. In addition, for the irregular shape of the metal magnetic powder, especially the sharp corners of the magnetic powder, finding out how to ensure the uniformity of the coating is also the key to the preparation process. Therefore, it is necessary to ensure that the magnetic powder matrix is uniformly coated by MgO-insulating material with excellent high-temperature-resistance, and the appropriate amount of addition should be considered to reduce the damage to the magnetic properties of the matrix [12].

In this investigation, high-temperature-resistant nano-MgO particles were employed as an insulation coating material, while phenolic resin served as an adhesive to enhance the bond strength between the substrate and the insulation layer. This approach was designed to ensure the adequate mechanical strength of the coating layer. The impact of MgO insulation material content on the microstructure and magnetic properties of soft magnetic composites was systematically examined. The results of this study provide a reference for the development and optimization of the preparation process and the properties of soft magnetic composites. Iron-based soft magnetic composites are useful in various applications, such as molding inductors and so on [13].

## 2. Experimental Materials and Methods

In this experiment, irregular reduced iron powder with a particle size of 200 μm, 99% Fe, 0.1% Si, and 0.3% Mn was used as the magnetic matrix. The material was provided by Changsha Tianjiu Metal Materials Co., Ltd., Changsha, China; The Xinhu Metal Materials Co., Ltd., Xingtai, China, provided the nano MgO particles for the insulating material, and the particle size was 50–100 nm and the purity was greater than 99.99%. Henan Hengyuan New Material Co., Ltd., Zhengzhou, China, provided the phenolic resin with a solid content not less than 70%. Nanjing Chuangde Chemical Additives Co., Ltd., Nanjing, China, provided coupling agent KH550. Tianjin Meiou Chemical Reagent Co., Ltd., Tianjin, China, provided the mold wall lubricant zinc stearate. Luoyang Chemical reagent factory, Luoyang, China provided the xylene solvent.

First, 100 g of reduced iron powder and KH550 were surface coupled at a mass ratio of 1:100. Then, the phenolic resin was added to the xylene solvent (the mass ratio of phenolic resin to MgO was 1:4), stirred until the resin was completely dissolved, and the coating suspension was prepared by adding 1 g, 2 g, 3 g, 4 g, 5 g, and 6 g of MgO, respectively. Finally, the iron powder after the surface coupling treatment was added to the above solution, and the coated iron powder was obtained by stirring and drying under the conditions of a 50 °C water bath. The coated iron powder was pressed into a ring of Φ40 mm × Φ32 mm × 4 mm under a pressure of 500 Ma for 60 s, and then the sample was heat-treated in a tubular sintering furnace (T1280A, Henan Chengyi Experimental Equipment Co., Ltd., Zhengzhou, China) in an argon atmosphere, heated to 500 °C, held for 1 h, and cooled to room temperature with the furnace.

Scanning electron microscopy (SEM) (Phenom XL100) was used to analyze the microstructure of the reduced iron powder and the shaped sample before and after the insulation coating. The insulating coating effect of the soft magnetic composite was characterized using an energy dispersive spectrometer (EDS). The density of the sample was determined using the Archimedes drainage method. The resistivity of the sample was measured using the four probe method. Under the condition of Bm = 50 mT, the magnetic permeability and magnetic loss of the materials in different frequency ranges were measured by using a soft magnetic AC analyzer (TD8120, Changsha Tianheng Co., Ltd., Changsha, China). The mechanical crushing strength of the sample was tested on a CMT 4305 electronic universal test machine.

## 3. Results and Discussion

### 3.1. Characterization of Insulation Layer

**Figure 1:**
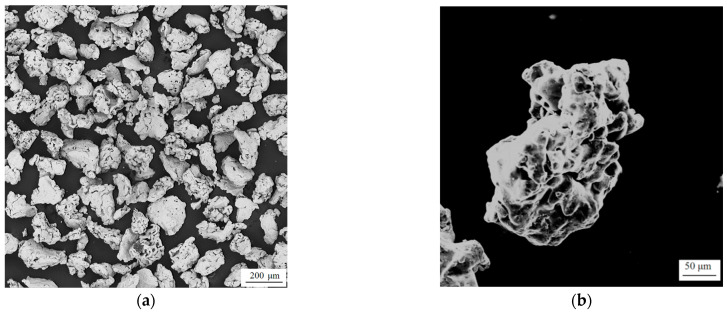

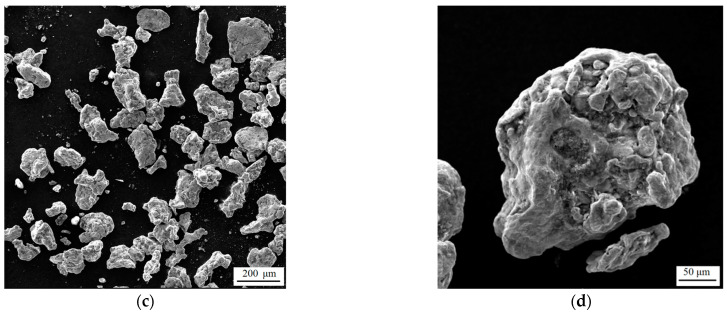
SEM image of magnetic particle surface: (**a**,**b**) show pure iron powders; (**c**,**d**) show insulated iron powders.

The surface morphology of reduced Fe powder before and after insulation coating is presented in Figure 1. Figure 1a–d, respectively, display SEM images of the untreated reduced Fe powder and the Fe powder after applying a 5 wt.% MgO coating at various magnification levels. Observing this Figure, it is clear that, in contrast to the as-received reduced Fe powder, the surface of the Fe powder treated with the insulation coating becomes relatively rough. The pores are decreased in size, and fine particles are observed adhered to the surface. This observation suggests the formation of an insulating coating layer on the surface of the reduced Fe powder matrix.

**Figure 2:**
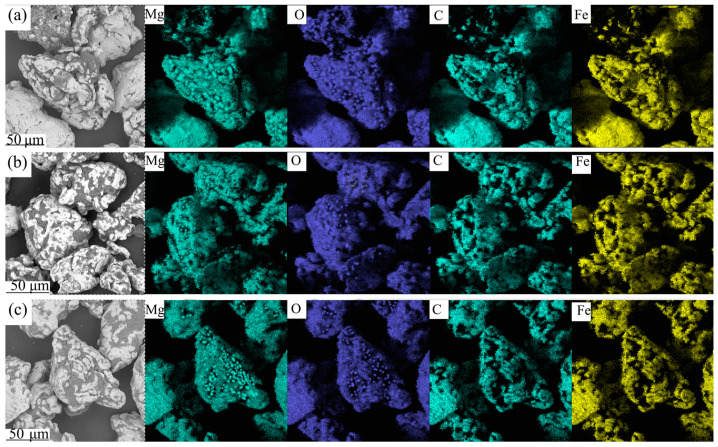
Element distribution maps of MgO-insulating iron powders: (**a**) 2 wt.% MgO; (**b**) 4 wt.% MgO; (**c**) 6 wt.% MgO.

The distribution of MgO on the surface of the Fe powder can be accurately determined by examining the elemental composition of the selected area. Figure 2 illustrates the surface elemental distribution of the Fe powder after insulation coating, with Figure 2a–c showing the results for 2 wt.%, 4 wt.%, and 6 wt.% MgO-coated magnetic powders, respectively. The energy spectrum scanning analysis reveals that Mg, O, C, and Fe elements are present on the surface of the Fe powder. This finding confirms that the surfaces of the Fe powder’s particles are encapsulated with an MgO insulation layer. Additionally, the distribution of the C element indicates that the phenolic resin effectively enhances the adhesion between the nano-MgO particles and the Fe powder, facilitating the uniform coverage of MgO on the Fe powder surface.

**Figure 3:**
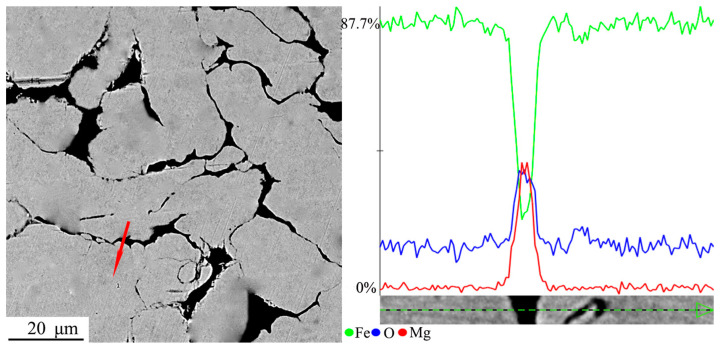
SEM micrograph of MgO-insulated compact body and atomic percentage line map of iron, magnesium, and oxygen elements. (Line scan in the direction of the arrow).

To further confirm the effectiveness of the insulation coating material, a local section of the magnetic ring pressed using Fe powder after insulation coating was sanded and polished. An energy spectrum line scanning analysis was then performed on the selected area near the interface of the magnetic powder particles. The distribution of elements within and between particles was evaluated using energy spectrum analysis, and the results are presented in Figure 3. The peak intensity of Fe elements is pronounced within the interior of the Fe particles, but it begins to diminish significantly as one moves towards the edges of the particles. In contrast, the peak intensity of Mg and O elements increases. When the peak intensity of Mg and O is compared to that of Fe, it is evident that the intensity of Mg and O is significantly lower than that of Fe. This observation suggests that the surface of the Fe powder is coated with a thin layer of MgO insulation, with Mg and O elements located between the Fe powder particles. This finding further validates the presence of an insulating coating on the surface of the Fe powder.

### 3.2. Effect of MgO Content on Material Properties

Table 1 lists the pressed density and resistivity of samples with varying amounts of MgO. The data indicate that, as the MgO addition increases, the density of the sample initially rises and then decreases. When 1 wt.% of MgO is added, the maximum value of the density is 6.57 g/cm^3^. However, when the MgO addition exceeds 4 wt.%, the density of the sample starts to decline sharply. Conversely, adding a small amount of MgO increases the density of the material because the MgO fills the spaces between the magnetic particles and enhances the bonding force between the MgO particles and the Fe powder. This improves the pressing performance. Nevertheless, as more MgO is added, the insulation layer thickens, the proportion of the ferromagnetic phase diminishes, and the density of the material gradually decreases. Following MgO coating, the resistivity of the sample is significantly enhanced. Specifically, the resistivity of the sample with 4 wt.% MgO is nearly 45 times greater than that of the sample without MgO under the same conditions. This substantial rise in resistivity demonstrates that the MgO coating effectively prevents direct contact between the iron powder particles, thereby minimizing the eddy currents induced by the external alternating magnetic field and reducing eddy current losses.

**Figure 4:**
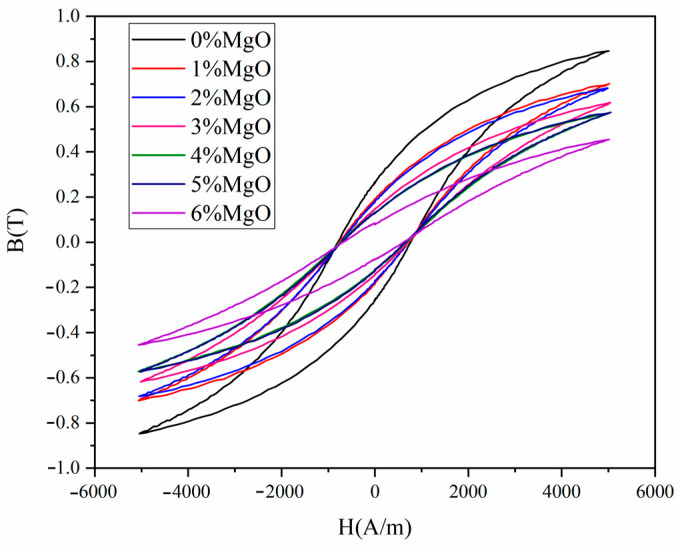
Hysteresis loops of MgO materials with different content.

Table 1 and Figure 4 shows the influence of different contents of MgO and resin on the DC properties of the material. With the increase in MgO and resin content, the initial permeability, maximum permeability, and saturation magnetic induction of the material showed a decreasing trend. This is because when using MgO and phenolic resin as coating materials, the two being in a non-magnetic phase, their presence will reduce the proportion of this magnetic phase.

**Figure 5:**
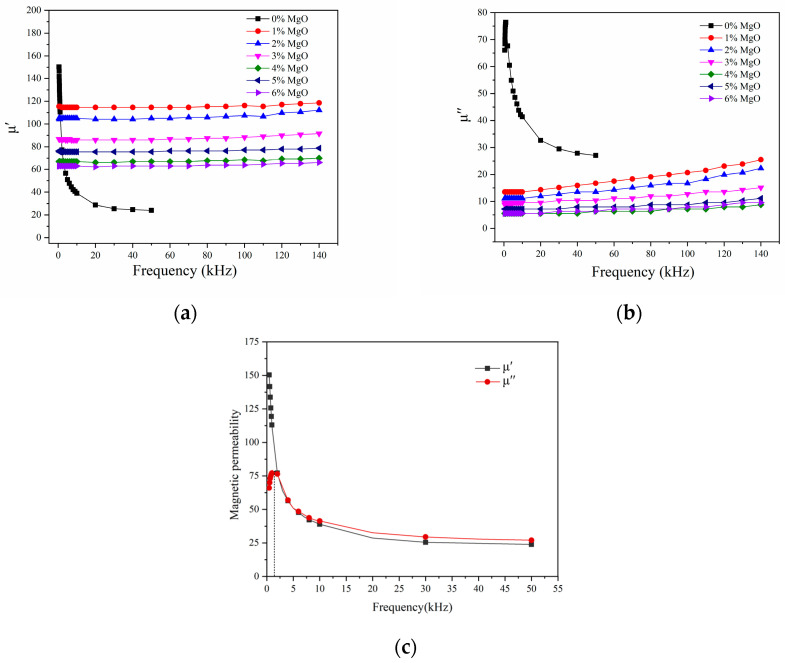
Complex permeability of soft magnetic composites at different frequencies: (**a**) real permeability μ′; (**b**) imaginary permeability μ″; (**c**) real and imaginary part values of the uncoated sample.

In an alternating magnetic field, the dynamic magnetic properties of ferromagnets are typically characterized by complex permeability, which includes both real and imaginary parts. The real part is related to the energy density stored in the magnetic medium, while the imaginary part represents the magnetic loss due to the magnetization state lagging behind the changes in the applied magnetic field [14]. Figure 5 illustrates the impact of MgO coating content on the complex permeability of a sample in the frequency range of 0.4–140 kHz. As can be seen in Figure 5a, the real part of the permeability of the uncoated sample at the initial measurement frequency is relatively high. However, it decreases rapidly with increasing frequency, reaching 150.4 at 1 kHz and only 23.8 at 50 kHz. After applying an MgO coating, the real part of the permeability of the sample remains relatively constant throughout the entire measurement frequency range, and the frequency stability is significantly improved. This is attributed to the MgO coating, which prevents direct contact between iron powder particles. As a result, the effective particle size is reduced, the eddy current loss between particles is decreased, and the permeability remains high at high frequencies [15].

The small eddy current loss in the soft magnetic composite prepared with MgO insulation coating indicates a larger skin depth, which allows the composite to operate at a higher frequency. Furthermore, as the content of non-ferromagnetic MgO insulation material increases, the air gap in the sample and the demagnetization field in the magnetic circuit are also increased, which hinders the magnetization process. Consequently, the real part value of the permeability of the sample decreases with the increase in the MgO coating amount. Figure 5b,c shows that the imaginary part value of the complex permeability of the uncoated sample has a peak value at a frequency of 2 kHz, while the imaginary part value of the complex permeability of the MgO-coated sample continues to rise with the frequency increase. At 50 kHz, the virtual part values of the permeability of the sample containing 1–6 wt.% MgO are 16.71, 13.52, 10.34, 6.36, 7.95, and 6.366385, respectively. At 100 kHz, the virtual part values of the permeability of the sample containing 1–6 wt.% MgO are 20.69075, 16.71, 12.73, 7.16, 8.75, and 7.95, respectively. Compared with other coating samples, The re-permeability of the sample containing 4 wt.% MgO had the lowest imaginary part value.

Increasing the content of the insulating agent can effectively increase the effective contact area between the iron powder particles and the insulating agent, which is beneficial for improving the resistivity of the sample and reducing the effective particle size. This leads to a reduction in eddy current loss in the magnetic powder core [16]. However, it should be noted that an excessive increase in insulating agent content may not always be beneficial. Higher insulating agent content can reduce the density and coercivity of the magnetic powder core, leading to increased hysteresis loss [17]. Therefore, the sample with 4 wt.% MgO coating amount has the lowest imaginary part value of complex permeability and a relatively acceptable real part value, making it the most suitable choice.

Formula (1) is the calculation of core loss by H Shokrollahi [2] and Jordan H [18] based on the improved formula of the Steinmetz equation:

(1) $$P_{S}=P_{h}+P_{e}=k_{2}B_{m}^{2}f+\frac{k_{3}B_{m}^{2}d^{2}}{\rho }f^{2}$$

where $k_{2}$ is the hysteresis loss factor and $k_{3}$ is the eddy current loss factor.

**Figure 6:**
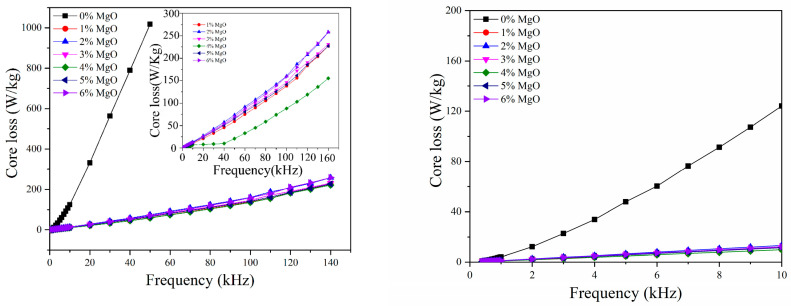
Core loss versus frequency for uncoated and MgO insulated compacts.

As shown in Figure 6, the core losses of both uncoated and MgO-coated samples in the frequency range of 0.4–140 kHz are depicted. The data reveal that the magnetic loss of the MgO-coated sample is significantly lower than that of the original powder sample. With the increase in frequency, the magnetic loss reduction becomes more pronounced. In comparison to the uncoated sample, the magnetic loss of the 4 wt.% MgO-coated sample was reduced by a factor of 77.3% at 1 kHz and by a factor of 94.2% at 50 kHz. This is attributed to the creation of a layer of MgO-insulating film on the surface of the iron powder, which results in a smaller effective grain size and higher resistivity. These factors collectively minimize the eddy current loss, leading to a low magnetic loss at high frequency for the soft magnetic composite.

In order to further study the effect of insulation coating content on the magnetic loss of soft magnetic composites, the loss separation formula was used to study the magnetic loss. The magnetic loss of the soft magnetic composite is composed of hysteresis loss, eddy current loss, and residual loss. The residual loss is composed of relaxation loss and resonance loss, which is important only at a very low magnetization field and high frequency, and can be ignored in the application of soft magnetic composite materials. Therefore, the total loss factor can be expressed as [19]:

(2) $$\tan\delta_{tot}=\tan\delta_{h}+\tan\delta_{e}=k_{2}+k_{3}f$$

**Figure 7:**
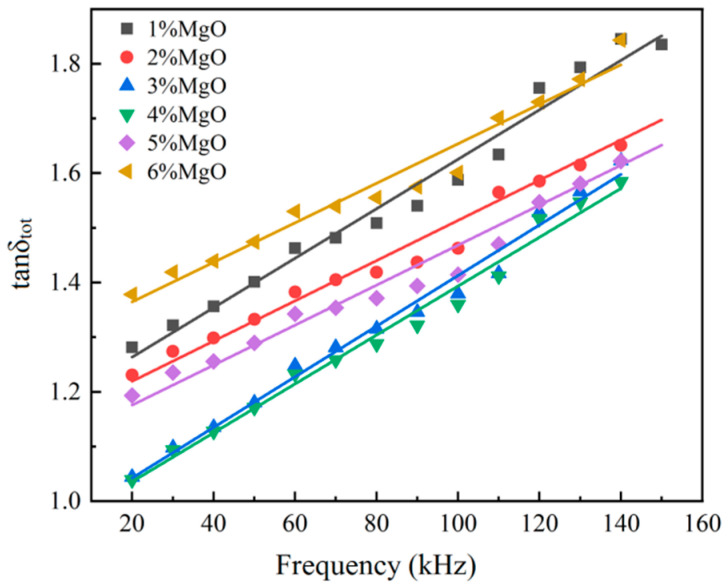
The variations in the magnetic loss factor with the frequency for the MgO-insulating samples.

Figure 7 presents a variation curve of the magnetic loss factor with the frequency for samples with various MgO-coating contents. It can be seen from the curve that magnetic loss factor and frequency are almost linear, the slope of each curve corresponds to eddy current loss factor ($k_{3}$), and the intercept of the straight line corresponds to the hysteresis loss factor ($k_{2}$). The values of the hysteresis loss factor and eddy current loss factor of the sample obtained using curve fitting are shown in Table 2. The following can be seen from the values in this Table: (1) The hysteresis loss factor ($k_{2}$) of the sample decreases first and then increases with the increase in MgO coating amount, and the value is the lowest when the MgO coating amount is 4 wt.%. Figure 3 shows that the high content of MgO leads to agglomeration. MgO not only fills the voids in the iron matrix, but also these clusters may be rich in iron. The isolation effect of MgO is poor, so the hysteresis loss increases, namely, the hysteresis loss coefficient is high [20]. (2) The eddy current loss factor ($k_{3}$) shows an overall decreasing trend with the increase in coating agent content. This is because the resistivity of the sample will be correspondingly increased with the increase in coating agent content (see Table 1). The eddy current loss of the sample is inversely proportional to the resistivity, so the eddy current loss factor of the sample decreases as a whole. Based on the above analysis and discussion, it can be seen that the 4 wt.% MgO-coated sample has the lowest hysteresis loss factor and a relatively low eddy current loss factor, and it can be determined that the optimum addition is at 4 wt.% MgO content.

**Figure 8:**
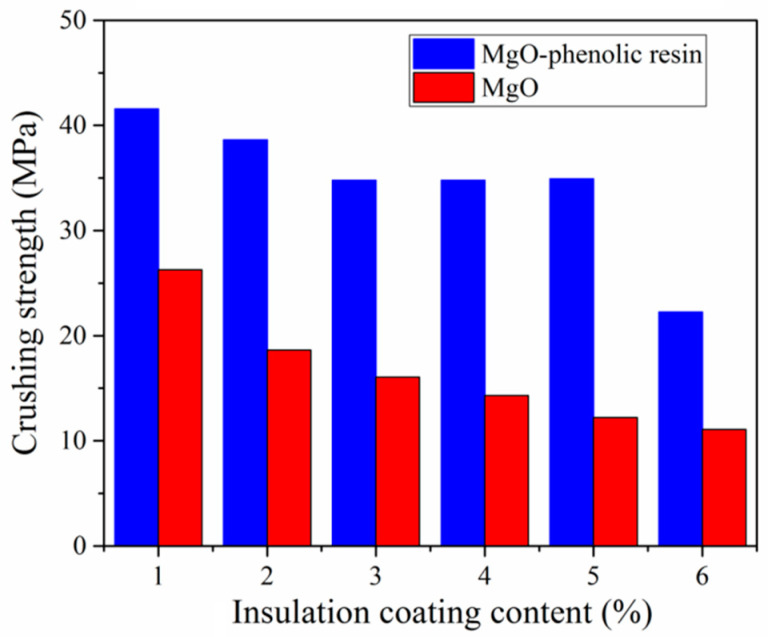
Comparison of MgO-phenolic resin and single MgO-coated samples in terms of the crushing strength.

Figure 8 shows a comparison of MgO-phenolic resin and single MgO-coated samples in terms of the crushing strength. As can be seen, the crushing strength of the sample with phenolic resin as a binder is higher than a single MgO coating sample, and the value increased from 14.3 MPa to 34.8 MPa when the amount of coating agent was 4 wt.%. The main reason for the increase in crushing strength is that the presence of adhesive phenolic resin can build an effective bridge between the matrix and the MgO nano-particles, increasing the bond strength between the insulating layer and the iron particles.

## 4. Conclusions

Soft magnetic composites were prepared by pressing and heat treatment with high-temperature resistant nano MgO as the insulating coating material and phenolic resin as the adhesive. The effects of different MgO contents on the properties of soft magnetic composites were studied. The conclusions are as follows:

1. The SEM and EDS analyses of the elemental surface distribution and line scanning showed that a layer of MgO insulation was uniformly coated on the surface of iron powder, and the presence of the insulation layer could effectively improve the frequency characteristics and resistivity of the sample. The resistivity of the sample coated with 4 wt.% MgO was nearly 45 times higher than that of the sample uncoated under the same conditions.
2. The coating agent content has an important influence on the magnetic properties of the sample. When the MgO content is 4 wt.%, the sample has an acceptable real permeability value and the lowest magnetic loss. Compared with the uncoated sample, the magnetic loss of the coated sample with 4 wt.% was reduced by 77.3% at 1 kHz. At 50 kHz, the magnetic loss is only 5.8% of the uncoated sample. The adhesive phenolic resin builds an effective bridge between the matrix and the MgO nano-particles, increasing the crushing strength from 14.3 MPa to 34.8 MPa when the coating amount is 4 wt.%.

## Figures and Tables

**Table 1:** Density and resistivity of samples with different MgO contents.

Sample	Density (g/cm^3^)	Electrical Resistivity (μΩ·m)
0 wt.% MgO	6.45	0.98
1 wt.% MgO	6.57	37.95
2 wt.% MgO	6.48	39.37
3 wt.% MgO	6.42	42.42
4 wt.% MgO	6.39	43.52
5 wt.% MgO	6.21	45.38
6 wt.% MgO	6.12	47.67

**Table 2:** The hysteresis factor *k*_2_ and eddy current loss factor *k*_3_ of the MgO-insulating samples.

Sample	*k*_2_	*k*_3_
1% MgO	1.17	0.0045
2% MgO	1.15	0.0037
3% MgO	0.95	0.0046
4% MgO	0.94	0.0044
5% MgO	1.10	0.0037
6% MgO	1.29	0.0036

## Data Availability

Data are contained within the article, further inquiries can be directed to the corresponding author.
